# Editorial: Health and physical literacy interventions in education, sport, and public health settings

**DOI:** 10.3389/fspor.2023.1353743

**Published:** 2024-01-08

**Authors:** João Martins, Marcos Onofre, Dean Dudley

**Affiliations:** ^1^ Centro de Estudos em Educação, Faculdade de Motricidade Humana e UIDEF, Instituto de Educação, Universidade de Lisboa, Lisboa, Portugal; ^2^ Macquarie School of Education, Macquarie University, Sydney, NSW, Australia

**Keywords:** health literacy, physical literacy, physical activity, healthy lifestyle, preventative health

**Editorial on the Research Topic** Health and physical literacy interventions in education, sport, and public health settings

## Introduction

Health Literacy (HL) and Physical Literacy (PL) are learning constructs that serve as the foundation for empowering all individuals and societies to adopt, maintain, and enjoy active, healthy, and sustainable lifestyles (1). Until recently, some HL and PL interventions have been applied to the fields of education, sport, and public health however may have been completed with insufficient rigor or oversight. In this context, Cairney and colleagues (2) proposed an evidence-informed conceptual model linking PL, emphasizing the need for empirical research and knowledge advancement. Following intense conceptual discussions on PL definitions (3), it's time to progress in the measurement, practical approaches, and field interventions (4). This was the motivation behind establishing this Research Topic, which includes seven papers that address these issues.

## Summary of selected articles from this research topic

Some articles contributed specifically to extending the HL and PL measurement field, helping to move beyond reductionist and decontextualised measures originally not created with the rationale of PL. Mota et al. presented the development and initial validation of the Portuguese Physical Literacy Assessment—Observation (PPLA-O) for Portuguese adolescents (15–18 years), which is one of the two components of the PPLA. The PPLA-O consists of two modules: Health-Related Fitness, and Movement Competence, Rules, and Tactics. The tool integrates data from physical education teachers and is designed for routine use to provide feedback to students and inform pedagogical decisions. The authors suggest that future research should focus on its reliability and validity.

In 2023, Mota et al. presented the Portuguese Physical Literacy Assessment (PPLA) validation for adolescents. This instrument includes the PPLA-O and the PPLA-Questionnaire. The PPLA tool was valid and reliable for assessing physical literacy as a multifaceted concept among Portuguese students aged 15–18 in a physical education context. This conclusion is consistent regardless of whether the analysis is based on individual factors or a composite score. Therefore, this instrument might be used to understand the PL levels of Portuguese adolescents who attend secondary school physical education.

Also, within the context of innovative PL measures, the study of Wilkie et al. developed and validated an observational tool for assessing children's PL in game-based physical education through an ecological dynamic rationale. This tool, which includes nine ecological conceptualizations of behavior, 15 measurement variables, and 44 categorical observational items, was valid and reliable, providing a valuable mechanism for educators and researchers to assess PL during gameplay.

The innovation of measurements is critical to understanding individuals' PL and its relationship with diverse literacies. Indeed, one study focused on the understanding and interconnection of diverse literacies in young people's lives. Bremer et al. conducted a cross-sectional study on Canadian children's attitudes towards various literacies, including reading/writing, math, and movement. The study sheds light on the children's consistent preference for physical activity in different social settings, such as school, home, and with friends. As a result, the study proposes potential approaches to improving PL and reducing gender inequalities in these contexts. For extending HL and PL knowledge, further studies should explore the meaning and interaction of diverse literacies in diverse contexts outside of school, such as sport and public health.

The work of Pushkarenko et al. brings critical considerations about individuals experiencing disability and the existing ableist PL narrative. Specifically, this paper critically examines PL discourse and practice, highlighting that current understandings often marginalize individuals with disabilities due to ableist norms. It is argued that while PL is intended for all, it is often operationalized as a privilege for some. The paper calls for adjusting the pedagogical approach to PL, offering practical recommendations to enhance inclusivity and provide meaningful experiences for all, which will help foster life-long physical activity engagement for all.

Moving this discourse even more into practice and intervention, Matthews et al. evaluated the impact of a virtual adapted physical activity program on the PL and activity of individuals with disabilities. Despite no significant increases in PL, the lack of decline during the pandemic (except in the cognitive dimension) was a promising finding. Future research should, therefore, explore the influence and effectiveness of virtual-based PL programs on individuals with disabilities and improve those intervention proposals.

The intervention protocol, led by Carl et al., aimed to develop and evaluate a PL intervention (“PLACE”) for third and fourth-grade children in the German all-day schooling system. The intervention protocol, which included 12 diverse sessions of 60–90 min each, is designed with a solid link to PL theory. The study has three phases, including two pilot studies and a main study. The results will determine the intervention's effectiveness and potential for scale-up and broader implementation and, therefore, to advance the field of PL and HL research, particularly in Germany.

## Future perspectives

The articles within this Research Topic of Frontiers in Sport and Active Living continue to add to the growing accepted realization that addresses PL, and now HL, are *learning constructs* influencing health-related behaviors. With this continued rising prominence of PL and HL in practical applications, following expert-driven templates designed to aid researchers in the strategic design and comprehensive documentation of interventions in these fields needs meticulous attention.

Whilst absent for HL, a recent publication template produced by Carl et al. (5) titled the *Physical Literacy Interventions Reporting Template (PLIRT)* is driving greater empirical rigor to be implemented in PL research. An adaptation of the PLIRT might also prove useful for HL interventions. The instrument comprises a comprehensive set of 14 items, with two additional items specifically tailored for studies utilizing mixed-methods approaches. These items are distributed across six distinct sections: title (one item), background and definition (three items), assessment (one item each for quantitative and qualitative studies), design and content (five items), evaluation (one item plus an additional item each for quantitative and qualitative studies), and discussion and conclusion (two items).

What is evident from this Research Topic on PL and HL intervention is that there needs to be enhanced transparency and interpretability of reports PL and HL interventions. Using expert-driven reporting templates such as the PLIRT for when publishing peer-reviewed research not only provides a useful writing schema for researchers, such tools are capable of bridging existing gaps between theoretical frameworks and practical applications, thereby contributing to the implementation of more comprehensive interventions within the realms of health, sport, and active living.
